# Atlantic salmon eggs favour sperm in competition that have similar major histocompatibility alleles

**DOI:** 10.1098/rspb.2008.1257

**Published:** 2008-10-14

**Authors:** Sarah E. Yeates, Sigurd Einum, Ian A. Fleming, Hendrik-Jan Megens, René J.M. Stet, Kjetil Hindar, William V. Holt, Katrien J.W. Van Look, Matthew J.G. Gage

**Affiliations:** 1 School of Biological Sciences, Norwich Research Park, University of East AngliaNorwich NR4 7TJ, UK; 2 Department of Biology, Norwegian University of Science and Technology, NO-7491Tungasletta 2, 7485 Trondheim, Norway; 3 Ocean Sciences Centre, Memorial University of NewfoundlandSt. John's, Newfoundland, Canada A1C 5S7; 4 Animal Breeding and Genomics Centre, Wageningen UniversityPO Box 338, 6700AH Wageningen, The Netherlands; 5 Cell Biology and Immunology Group, Wageningen Agricultural UniversityPO Box 9101, 6700 HB Wageningen, The Netherlands; 6 Institute of Zoology, Zoological Society of LondonRegent's Park, London NW1 4RY, UK

**Keywords:** major histocompatibility genes, sperm competition, cryptic female choice, fertilization, Atlantic salmon

## Abstract

Polyandry and post-copulatory sexual selection provide opportunities for the evolution of female differential sperm selection. Here, we examined the influence of variation in major histocompatibility (MH) class I allelic composition upon sperm competition dynamics in Atlantic salmon. We ran *in vitro* fertilization competitions that mimicked the gametic microenvironment, and replicated a paired-male experimental design that allowed us to compare differences in sperm competition success among males when their sperm compete for eggs from females that were genetically either similar or dissimilar at the MH class I locus. Concurrently, we measured variation in spermatozoal traits that are known to influence relative fertilization success under these conditions. Contrary to the findings demonstrating mechanisms that promote MH complex heterozygosity, our results showed that males won significantly greater relative fertilization success when competing for eggs from genetically similar females at the MH class I. This result also showed covariation with the known influences of sperm velocity on relative fertilization success. We discuss these unexpected findings in relation to sperm–egg recognition and hybridization avoidance mechanisms based upon immunogenetic variation.

## 1. Introduction

Biologists now know that sexual selection operates after mating to the level of the gamete (Parker 1970), where forces and adaptations recognized at the whole organism level for male–male competition and female choice can be paralleled by adaptations for sperm competition and cryptic female choice (Eberhard 1996; Birkhead & Moller 1998). Evidence for cryptic female choice at the gamete level has been challenging to obtain, because it is difficult to disentangle the influence of spermatozoal adaptations from female effects (Birkhead 1998), especially in internal fertilizers. However, non-confounded examples of female differential sperm selection have been identified in a number of systems (e.g. Pizzari & Birkhead 2000; Pilastro *et al*. 2004). Some of the clearest demonstrations of cryptic female choice have been discovered in external fertilizers, where gamete interactions can be controlled and measured in a natural fertilization microenvironment. One of the first examples of sperm discrimination was recorded under self/non-self recognition in tunicates: sperm carrying identical alleles at a ‘fusibility’ locus were less compatible for egg penetration (Scofield *et al*. 1982). In the hermaphroditic ascidian *Diplosoma listerianum*, cryptic female choice of sperm allows ova to avoid self-fertilization through discrimination against genetically similar sperm (Bishop *et al*. 1996). By contrast, cryptic female choice in the opposite direction against genetically different sperm has also been demonstrated. In sea urchins (*Echinometra* sp.), external fertilization is mediated by attachment to the egg of the sperm protein bindin (reviewed in Vacquier *et al*. 1995). Palumbi (1999) showed that in the sea urchin *Echinometra mathaei*, eggs select sperm with a bindin genotype similar to their own, as a possible mechanism to avoid hybridization and preserve local adaptation.

Mechanisms of cryptic female choice by the egg have therefore evolved. However, a particular difficulty in proving cryptic female choice has been isolating male effects from female influence at the level of the gamete (Birkhead 1998). Under internal fertilization, where the majority of sperm competition and cryptic female choice experiments have been conducted, male post-copulatory effects could override female effects. If male influences on ejaculate transfer, migration, retention and sperm traits are not controlled for, then any female effect on fertilization dynamics could be confounded by these recognized male influences. In this study, we build on previous work (Gage *et al*. 2004) to explore cryptic female choice within the external fertilization system of the Atlantic salmon *Salmo salar* by conducting controlled *in vitro* sperm competition experiments, while measuring spermatozoal traits known to explain differential fertilization success.

Atlantic salmon are typically polyandrous under natural spawning situations (Fleming 1996), leading to post-copulatory sexual selection on males and females. By running *in vitro* sperm competition experiments in parallel to detailed sperm screening (of relative number, motility, longevity and size), both under conditions that mimic the gamete microenvironment, we have demonstrated that relative sperm velocity is an important gametic predictor of sperm competitiveness (Gage *et al*. 2004). With this predictive information, we can run similar experiments, monitoring spermatozoal traits, to test for cryptic female choice at a gene with important fitness consequences: the major histocompatibility (MH) class I locus.

The vertebrate MH complex (MHC) is a large cluster of genes involved in the acquired immune response and immunological self/non-self recognition. It includes the most variable genes known to date in vertebrates, with up to 349 alleles described for a single locus (Robinson *et al*. 2000) and heterozygosity values greater than that would be predicted by neutrality (Edwards & Hedrick 1998). MH genes encode cell surface glycoproteins responsible for the recognition and presentation of foreign antigens to immune effector cells (e.g. Potts & Wakeland 1990). MH genes incorporate two main classes, classes I and II, each consisting of two or more loci (Klein 1986). Class I molecules are found on nearly all cell types and present intracellular peptides to T cells, while class II is found on only a few specialized cell types and present peptides derived from extracellular proteins (e.g. Potts & Wakeland 1990; Jeffery & Bangham 2000); we therefore focus our tests on MH class I recognition. Peptide-binding specificity, T-cell response and therefore immune system function are dependent on the highly polymorphic nature of MH genes, particularly at the peptide-binding site (reviewed in Potts & Wakeland (1990); Jeffery & Bangham (2000); Piertney & Oliver (2006)). This association between the immune response and MH polymorphism suggests that infectious diseases are one of the main selective forces that drive and maintain the extraordinary diversity of MH genes (Doherty & Zinkernagel 1975). However, the extreme variability of these genes may also be maintained via MH-based mating preferences where choice for MH-dissimilar mates promotes offspring MH genetic diversity (Jeffery & Bangham 2000).

A number of studies have documented mate choice in relation to MH genes, with females promoting offspring MHC diversity through preferences for MHC-dissimilar male mates (e.g. mice, Potts *et al*. 1991; Penn & Potts 1998), humans (Wedekind *et al*. 1995; Ober *et al*. 1997; Wedekind & Furi 1997) and birds (Freeman-Gallant *et al*. 2003; Richardson *et al*. 2005; Bonneaud *et al*. 2006). Studies in fishes have shown promotion of diversity through intermediate MHC dissimilarity (Reusch *et al*. 2001; Forsberg *et al*. 2007). There is evidence for MH-disassortative mating preference in Atlantic salmon. Molecular parentage studies of a closed but naturally spawning salmon population showed that a greater level of offspring heterozygosity at MH genes was produced than would be predicted by random mating (Landry & Bernatchez 2001; Landry *et al*. 2001). Two recent studies also showed that MH class II genes in Atlantic and Chinook salmon showed greater dissimilarity among offspring batches than random mating would predict, suggesting that disassortative mating for MH-different partners or fertilization occurred (Consuegra & Garcia de Leaniz 2008; Neff *et al*. 2008). We therefore explored whether such female choice of MH genes within a polyandrous mating system could occur at the level of the gamete through cryptic female choice within the potential fertilization set.

There is evidence that human spermatozoa express their own haplotypic variation at the MHC (e.g. Arnaizvillena & Festenstein 1976), but there are also studies finding no such effect (e.g. Haas & Nahhas 1986). Wedekind *et al*. (1996) provided a helpful synthesis of the differing findings, suggesting that MHC expression may be condition dependent, and therefore variable through time. In mice, which demonstrate clear MHC-based mate choice (Yamazaki *et al*. 1976), there is also some evidence to suggest MHC selectivity at the gamete level. Wedekind *et al*. (1996) used congenic lines, differing only at the MHC, to demonstrate an MHC influence upon *in vitro* fertilization compatibility, but the effect was not consistent over time. Similar *in vitro* experimental testing was performed using externally fertilizing whitefish (*Coregonus* sp.), employing a split-family design, and although there was an MHC class II effect on disease resistance, no MHC influence was detected on relative fertility (Wedekind *et al*. 2004). However, in a recent study, Skarstein *et al*. (2005) showed in charr *Salvelinus alpinus* that males heterozygous at MHC class IIβ had higher fertilization success under *in vitro* conditions than homozygotes, suggesting positive fertilization compatibility for sperm from MHC II heterozygous males.

Of the very few studies so far conducted, evidence for MHC-dependent sperm selection is therefore variable. In this study, we introduce a new factor to the exploration of MHC-dependent sperm selection: sperm competition. Unless conditions are sperm limited, it is possible that mechanisms of cryptic female choice will only be detectable under polyandry, when females are provided with a ‘choice’ of different males' sperm. We replicate a reciprocal paired-design experiment, where we competed sperm from two salmon for a female's eggs under controlled *in vitro* conditions. The female is genetically similar to male A at the MHC class I locus, but dissimilar to male B. We then switch the female, and competed the same male pair again for eggs from a new female, which is now similar to male B, but dissimilar to male A. By concurrently assessing spermatozoal traits in the environment to which gametes are naturally adapted (Gage *et al*. 2004), and assigning paternity using microsatellite markers, we can determine the variance in sperm competition success that is attributable to MHC dissimilarity, and therefore cryptic female choice.

## 2. Material and methods

### (a) MHC typing and experimental fish groups

Sperm competition trials and egg rearing were performed at the Norwegian Institute of Nature Research (NINA) Research Station, Ims, southwestern Norway. Sea-ranched anadromous adults were caught in a fish trap located 100 m above the outlet of the River Imsa, close to NINA Research Station. All fish had been tagged at release as smolts, and therefore known to be derived from the Imsa population. All animal experiments described were approved by the Norwegian Animal Research Authority.

Forty-one males and 59 females were anaesthetized and passive integrated transponder tagged. Small fin clips were taken and used to type the fish for MH class I genes using neutral microsatellite markers that amplify simple repeats embedded in these MH genes of Atlantic salmon. The length of these repeats is diagnostic for allelic variation in MH class I genes of Atlantic salmon (Grimholt *et al*. 2002).

DNA was extracted using the Promega Wizard DNA extraction kit, following the manufacturer's instructions. Typing was done based on the genomic DNA extracted from the fin clips according to Grimholt *et al*. (2002) and Consuegra *et al*. (2005). The microsatellite in the 3′-untranslated tail of the class I locus (*Sasa-UBA*) was PCR amplified using a fluorescently labelled forward primer (SasaUAgts2F 5′-AGGAAGGTGCTGAAGAGGAAC-3′) and a reverse primer (5′-CAATTACCACAAGCCCGCTC-3′). Genotyping was performed on an ABI prism 377 automated sequencer (Applied Biosystems).

To confirm the MH class I type of the individuals included in the experimental design, sequencing of the expressed MHC class I alleles for six of the parents was conducted at the end of the experiment when the fish could be killed. Anterior kidney samples were stored on RNAlater and RNA extraction was performed using the Promega SV total RNA isolation system. The mRNA extracted was used to generate cDNA using RT-PCR (Superscript one-step RT-PCR kit, Invitrogen) to amplify a region of approximately 500 bp (spanning the α1- and α2-domains) with a 50 : 50 mix of the following primers (Grimholt *et al*. 2002). For forward priming, Lead2S: 5′-CTGGGAATAGGCCTTCTACAT-3′ and Lead4S: 5′-AGCCCTACATTCTTCATC TGC-3′, and the reverse primer, UBAex3R: 5′-TGTCCTIATCAGAGTGCTCTT CC-3′.

The resulting RT-PCR products were purified and cloned into a *t*-tailed plasmid vector using the Promega PGEM T-easy cloning kit. Both strands of at least five clones per individual were sequenced with the ABI Prism BigDye Terminator Cycle Sequencing kit, and the sequences were resolved on an ABI Prism 377.

Using the MH typing results, two-male and two-female mate choice and fertilization compatibility combinations were ascertained. Eighteen males and eighteen females each were split into nine groups of four (two males and two females) such that female 1 was similar to male A but dissimilar to male B, and female 2 was dissimilar to male A but similar to male B, with respect to MHC class I alleles. For the similar crosses, males and females shared identical MH class I alleles; dissimilar crosses had different alleles.

### (b) *In vitro* sperm competitions and analysis of sperm form and function

Prior to conducting the fertilization trials, the fish were kept in 47 m^2^ circular stream-section arenas designed to simulate natural breeding conditions (as described in Fleming *et al*. 1996). When females showed gravel digging behaviour for spawning, they were caught and their eggs stripped; the timing of digging behaviour was independent of MH class I genotype. Individuals were lightly anaesthetized using chlorobutanol, and stripped of eggs using gentle abdominal pressure. The two experimental males (one MH similar and the other MH dissimilar) were gently stripped of milt without anaesthesia (which could have confounded sperm function (Wagner *et al*. 2002)) and the sperm samples evenly diluted into trout extender (Billard & Cosson 1992) at a 1 : 1 ratio. The use of extender prevents any potential pre-activation of the sample.

Approximately 50 eggs from each female were fertilized using 50 μl sperm/extender (1 : 1) from the two males. The sperm/extender samples from each male were separately mixed, and gametes placed on the opposite sides of a dry 1 l plastic beaker. Sperm and eggs were activated and mixed in the turbulence when 500 ml Ims River water at the natural river temperature (3(±1)°C) was rapidly added.

Sperm activity was recorded at 3(±1)°C within 30 min of fertilization using a Sony Hi8 tape deck connected to a JVC video camera (TK-1280E) fixed to an Olympus CK40 inverted stage microscope calibrated to a slide at 400× magnification under dark-field phase illumination. Subsamples of sperm in extender were activated by mixing milt in river water, and then 0.7 μl of the active diluent was immediately transferred onto a 12-well multi-test glass slide (ICN Basingstoke, UK) (well depth=∼0.0116 mm) and a cover-slip carefully and quickly put in place. Milt (3–6 μl) and river water volumes (350–400 μl) used for activation were adjusted, so that 50–100 spermatozoa were visible on the field of view at 400× magnification, and all sperm are simultaneously and evenly activated. The time of sample activation was recorded, and sperm activity was video recorded until sample death.

Sperm motion characteristics were analysed using a computer-aided sperm analysis system, the Hobson Sperm Tracker (Hobson Vision Ltd., Baslow, UK). The tracker was set to operate at a frame rate of 50 Hz and the ‘minimum track point’ setting was 50 frames. The ‘search radius’ used was 8.13–10.56 μm and the ‘threshold’ set to +30/−100 with the objective at 40×. Tracking was initiated 5 s after activation, allowing 3 s for activated sperm to be transferred to the slide, and 2 s for stabilization of the image. Tracking periods were set to 15 s, as this is the shortest time period in which the Hobson tracker can calculate the percentage of motile sperm. The most informative data come from the first tracking period, i.e. 5–20 s after activation (Kime *et al*. 2001). Sperm longevity is the period from activation until the sperm no longer show forward progressive motility. Swimming speed was calculated from individual sperm swimming tracks to determine average curvilinear velocity. Sperm density was also calculated from sperm counts on diluted subsamples from a haemocytometer (improved Neubauer chamber) according to the established protocols (Gage *et al*. 1998).

Eggs were reared at the NINA Station in uniquely coded egg trays placed in incubation channels with constant water flow. When eyed embryos became visible after 2–3 months, they were preserved in ethanol for genetic analysis. All egg batches had 100 per cent fertilization success, controlling for the effects of sperm limitation. There was negligible (less than 3%) egg mortality during the incubation period, also suggesting no MHC-based effects on egg development.

### (c) Microsatellite paternity analysis

Microsatellite genetic analysis was performed to determine parental genotypes and offspring paternity. Paternity of 30 offspring from each IVF competition was determined. A total of 36 adults and 540 embryos were screened. Tissue samples were placed in 96-well plates with 50 μl TEN buffer (400 mM NaCl, 10 mM Tris–HCl (pH 8), 2 mM EDTA (pH 8) and 2% SDS (9 : 1 ratio)) and 5 μl proteinase K (10 mg ml^−1^) and incubated at 50°C overnight. Fifteen microlitres of 6 M NaCl were added to each well before centrifugation at 3000*g*. Ten microlitres of supernatant from each well were transferred to a clean plate using a multi-channel pipette and 20 μl of 100 per cent cold ethanol added to each sample. The plate was then left at −80°C for at least 30 minutes before centrifugation at 3000*g* for 30 min. The supernatant was discarded and each DNA pellet washed with 150 μl of cold 70 per cent ethanol. The DNA pellets were then dried at 50°C for 20 min before resuspension in 100 μl water. The plates were left at 37°C for 30 min to ensure resuspension of the DNA, which was then stored at −20°C.

Paternity was analysed by surveying microsatellite variation at two pure loci (Ssa408 and Ssa410) and one compound locus (Ssa421) (Cairney *et al*. 2000). PCR amplification was carried out in a 10 μl volume containing 1 μl of template DNA (unspecified concentration), 5 μl of 2× PCR Master Mix with 1.5 mM of MgCl_2_ (Abgene), 1 μl of BSA (10 mg ml^−1^), 0.5 μM of labelled forward primer, 0.5 μM of reverse primer and sterile distilled water to total volume. Forward PCR primers were fluorescently labelled with FAM (Ssa408), HEX (Ssa410) and NED (Ssa421) (Applied Biosystems). An initial 3 min denaturation at 94°C preceded 29 denaturing (94°C for 15 s), annealing (*Χ*°C for 15 s) and extension (72°C for 15 s) cycles. Annealing temperatures were 58°C for Ssa408 and Ssa421 and 53°C for Ssa410.

PCR products were run on an ABI3700 automated DNA sequencer with the Genescan-500 ROX-labelled size standard (Applied Biosystems). Fragment lengths were determined using the GeneScan and Genotyper software packages v. 3.7 (Applied Biosystems). Once the parental genotypes were known, only one, occasionally two, of the specified loci was needed for unambiguous paternity exclusion of the embryos in each two-male competition. Parentage was determined by comparing alleles at the locus or loci used, with alleles from the mother and both of the potential fathers.

### (d) Statistical analyses

We employed a paired-male experimental design, so that relative fertilization success could be directly compared between different females when males were competing for fertilizations in both MH-similar and MH-dissimilar roles (in random order). Each two-male competition is therefore repeated, using a reciprocal MH genotype female (in random order), allowing comparison within one (randomly selected) male from each of the nine competing pairs using a paired *t*-test. To determine that results from our random choice of focal males in the paired test was representative of the overall distribution of all possible data, we also performed Monte Carlo resampling (999 iterations with replacement) of all possible relative fertilization success differences for each of the nine 2-male/2-female combinations using the Excel add-in PopTools v. 3.0 (www.cse.csiro.au/poptools/).

Having analysed for an effect of MH genotype using this controlled paired design, we then explored whether any MH-dependent relative fertilization success showed covariation with sperm traits known to be important in sperm competition using ANCOVA. Measurement of spermatozoal characteristics were repeated for each different fertilization trial, so that any within-male changes in sperm traits between trials (up to several weeks apart depending upon when the female came into reproductive condition) were individually controlled for. In the ANCOVA, we analysed across all 18 different females, so that our analytical unit of independence is an individual female's relative fertilization compatibility with one (randomly chosen) male in relation to MH similarity and the covariates of relative sperm velocity, density of motile sperm and sperm longevity (Gage *et al*. 2004). Relative sperm traits were calculated by dividing the focal male's sperm trait by his competitor's sperm trait. Finally, to ensure that our random choice of males was a fair representation of the actual distribution of any relationships within our dataset, we ran 100 repeats of the ANCOVA analyses if any variable showed an initial covariance significance of *p*<0.1, randomizing male inclusion for each of the 100 runs.

## 3. Results

Data distributions did not depart significantly from normality. Our results revealed a significant difference between individual male sperm competition success dependent on MH genetic similarity: males won approximately 15 per cent more fertilizations when competing for MH-similar females (paired *t*_8_=2.75, *p*=0.025; mean sperm precedence of males when MH similar was 45.4%±6.85 s.e. and when dissimilar was 32.3%±6.55 s.e.; figure 1). Monte Carlo iterations of all possible relative fertilization success differences showed that when males competed for MH-dissimilar females, they consistently achieved an average 13 per cent reduced relative fertilization success where the lower confidence limit did not breach zero (999 iterations: mean marginal difference in % fertilization success=−13.13%, lower confidence limit=−22.06%, upper confidence limit=−3.9%).

Importantly, this MH-based effect is evident while controlling for known influences of sperm velocity on sperm competition success (Gage *et al*. 2004). ANCOVA showed that relative fertilization success was dependent on MH genetic similarity (100 randomized ANCOVA repeats: average *F*_1,17_=4.92, *p*<0.05) while revealing a significant covariance with relative sperm curvilinear velocity (100 randomized ANCOVA repeats: average *F*_1,17_=8.86, *p*<0.02). There was no difference in average sperm velocity between MH treatments (100 paired *t*-test repeats, randomizing focal male inclusion: average paired *t*_8_=1.28 (maximum *t*=1.6), *p*=0.24 (minimum *p*=0.15)). No significant covariation was found between motile sperm density (*F*_1,17_=0.627, *p*=0.443) or sperm longevity (*F*_1,17_=1.081, *p*=0.317) and sperm competition success, in consistence with previous work under similar conditions (Gage *et al*. 2004).

## 4. Discussion

Our experiments showed that *in vitro* fertilization success was significantly greater when sperm from individual males compete to fertilize eggs from females that are genetically more similar at the MH class I locus in Atlantic salmon. This finding was confirmed after we controlled for the effects of sperm traits on the dynamics of sperm competition. The findings are contrary to theoretical and empirical arguments for the promotion of MH heterozygosity (Edwards & Hedrick 1998), and evidence showing behavioural preferences for MH-dissimilar mates or matings (e.g. Wedekind *et al*. 1995; Wedekind & Furi 1997; Penn & Potts 1998; Landry *et al*. 2001), including mechanisms driving MH heterozygosity surpluses in salmon (Landry *et al*. 2001; Consuegra & Garcia de Leaniz 2008; Neff *et al*. 2008). Our fertilization compatibility results reveal a mechanism at the gamete level that actually promotes MH class I homozygosity, so how can these counterintuitive results be explained?

The optimal outbreeding hypothesis (Bateson 1978) recognizes that there is an optimal genetic relatedness between mates that lies somewhere between kin (leading to inbreeding depression) and hybrid genes (leading to outbreeding depression). Lynch (1991) considered that inbreeding and outcrossing could even operate simultaneously in an individual's genome. Could such a situation occur in salmon, with enhanced selection for locally adapted MH genes, compared with outbreeding for other traits where heterosis is important? The MH relatedness spectrum across our source population of 100 males and females showed that the ‘MH-similar’ pairings (that achieved higher sperm competition success) were between males and females with genetically identical MH alleles, and therefore as genetically close at the MH locus as the population's allelic variation allowed. An optimum mate choice within a genetic spectrum has been revealed in sticklebacks, where females choose males that will generate an optimal number of MHC alleles in offspring genotypes (Reusch *et al*. 2001), which is neither the maximum nor the minimum in the population. However, in contrast to the stickleback MHC where multiple alleles can be expressed (Reusch *et al*. 2001), Atlantic salmon express a maximum of two alleles at the class I locus (Grimholt *et al*. 2002), so that any spectrum compatibilities will be limited to paired allelic variation under either homozygous or heterozygous states. Accordingly, the position of the optimum is constrained to a maximum of two male and two female alleles, giving four possible combinations.

In seeking an adaptive explanation, our results are consistent with a fertilization mechanism that has evolved to limit hybridization or outbreeding depression. In salmon, where most adults return to their natal site to spawn within relatively small geographical scales (Garant *et al*. 2000), local genetic adaptation is under strong selection (Taylor 1991), and risks from hybridization with trout are established (Garcia-Vazquez *et al*. 2001). Such a scenario within semi-isolated populations could theoretically shift the ‘outbreeding optimum’ closer to genetic relatives. Selection for local adaptation could therefore drive a mechanism that promotes reproduction between genetically more similar individuals. MH genetic mediation of such reproductive compatibilities would make even more sense if there was local adaptation to varying pathogen challenges. Such local adaptation by the MH class IIβ in relation to temperature cline and pathogen challenge has recently been shown in Atlantic salmon through a large and detailed survey of MH genetic variation across 1549 salmon in 34 river populations (Dionne *et al*. 2007). More specifically, evidence shows that resistance to *Aeromonas salmonicida* infection in Atlantic salmon drives selection pressures on specific alleles, rather than general selection for increasing MH heterozygosity (Lohm *et al*. 2002). A similar phenomenon has been experimentally demonstrated in Chinook salmon, *Oncorhynchus tshawytscha* (Pitcher & Neff 2006). There may therefore be positive selection for reproduction between individuals carrying a specific MHC genotype that is optimal for the population, and selection against dilution of this locally adapted genotype, thus explaining our MHC similarity fertilization advantage. Our experiment used adult salmon all marked and sampled from the same River Imsa population, so it seems unlikely that the lower reproductive compatibility in the MHC-dissimilar crosses is the result of a mechanism to avoid hybridization. However, it is possible that eggs (by comparison with adults) are unlikely to have evolved a sophisticated mechanism that selects for genetic compatibility somewhere within a continuous spectrum, so that the mechanism to preserve local adaptation or avoid hybridization is to be the most compatible with the most genetically similar gametes. This ‘similarity compatibility’ mechanism using immunorecognition could then be overridden at the whole animal level through more sophisticated mate-choice mechanisms.

If the MH genetic variation mediates gamete compatibility through local adaptation in Atlantic salmon, what mechanisms influence sperm–egg recognition? Gamete selection for similar genotypes has been clearly demonstrated in externally fertilizing species that do not have MH genes (e.g. Scofield *et al*. 1982; Palumbi 1999). In *Echinometra* sea urchins, external fertilization is mediated by attachment to the egg of the sperm protein bindin (reviewed in Vacquier *et al*. 1995) and, as the MHC, this protein is highly polymorphic within species (Metz & Palumbi 1996). Palumbi (1999) showed in *E. mathaei* that eggs ‘select’ sperm with a bindin genotype similar to their own, suggesting strong linkage between female choice and male trait loci. He also found that individual females differed in their sperm preferences but showed consistency. In parallel to MHC-based mate choice, there is no universal ‘best bindin’, but compatibility between specific male and female bindin genotypes. In a manner similar to these within-species studies, fertilization compatibility has also been detected and described between species to avoid hybridization. Different *Haliotis* abalone species occur under sympatry, yet natural hybrids are rare. A species-specific fertilization mechanism avoids hybridization by promoting a sperm competition advantage to the conspecific sperm through a cognate pair of gamete recognition proteins: sperm lysin and egg vitelline envelope receptor for lysine (VERL) (Swanson & Vacquier 1997; Galindo *et al*. 2003). The non-enzymatic sperm protein lysin binds to the VERL receptor on the egg vitelline envelope (Swanson & Vacquier 1997). Lysin–VERL binding and lysine-mediated vitelline envelope dissolution show species specificity (Swanson *et al*. 2001), so that sperm–egg compatibility is controlled by specificity between these two variable protein molecules (Swanson & Vacquier 1997).

Less is known about the mechanisms of sperm–egg interaction at fertilization in salmon. Gamete association after release into turbulent water in salmonids is rapid (Hoysak & Liley 2001), with a 2 s delay in sperm release into *in vitro* fertilization competitions having significant effects on the relative fertilization success of the delayed male's sperm (Yeates *et al*. 2007). This rapid sperm–egg association makes it difficult to identify a mechanism where MH gene selection by the ovum can occur. However, little is understood of what occurs after sperm enter the single egg micropyle in salmon, and how many sperm actually enter the egg. Although there is a fertilization block via a membrane potential change after sperm entry (Gilkey 1981), nothing is known of the mechanics of that egg membrane penetration, or if multiple sperm enter the ovum to seek the pronucleus. There is no evidence for pathological polyspermy: *in vitro* fertilizations using extreme sperm concentrations (50 : 50) show similar fertility and embryo survival as low dilutions (M. J. G. Gage 2000, unpublished data). Given the large internal volume of a salmon egg (diameter 5000–6500 μm) relative to the average length of a sperm (30–40 μm) and the micropylar canal 30 μm (Kobayashi & Yamamoto 1981), there may be further stages of sperm survival, competition and selection inside the ovum before pronucleus fusion, which have yet to be identified. Alternatively, differential discarding of the egg's second polar body after fertilization could provide a mechanism of fertilization choice (Wedekind *et al*. 1996; Pitcher & Neff 2006).

## Figures and Tables

**Figure 1:**
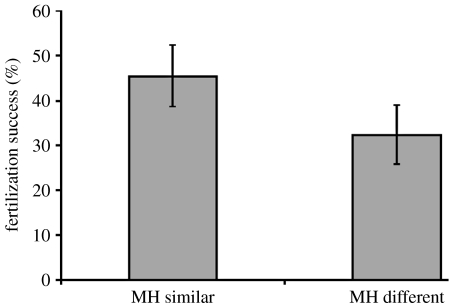
Mean fertility (% paternity) ±1 s.e. of nine different focal males competing in two-male sperm competition trials for eggs from nine MH-similar and nine MH-dissimilar females (paired *t*_8_=2.75, *p*=0.025).
